# High GFPT1 expression predicts unfavorable outcomes in patients with resectable pancreatic ductal adenocarcinoma

**DOI:** 10.1186/s12957-021-02147-z

**Published:** 2021-01-31

**Authors:** Yitao Gong, Yunzhen Qian, Guopei Luo, Yu Liu, Ruijie Wang, Shengming Deng, He Cheng, Kaizhou Jin, Quanxing Ni, Xianjun Yu, Weiding Wu, Chen Liu

**Affiliations:** 1 Department of Pancreatic Surgery, Fudan University Shanghai Cancer Center, No.270 DongAn Road, Shanghai, 200032 China; 2 Department of Oncology, Shanghai Medical College, Fudan University, Shanghai, 200032 China; 3 Shanghai Pancreatic Cancer Institute, Shanghai, 200032 China; 4 Pancreatic Cancer Institute, Fudan University, Shanghai, 200032 China

**Keywords:** Glutamine-fructose-6-phosphate transaminase 1, Hexosamine biosynthesis pathway (HBP), Survival, Prognosis

## Abstract

**Background:**

Glutamine-fructose-6-phosphate transaminase 1 (GFPT1) is the first rate-limiting enzyme of the hexosamine biosynthesis pathway (HBP), which plays a pivotal role in the progression of pancreatic ductal adenocarcinoma (PDAC). Therefore, we investigated the prognostic significance of GFPT1 expression in patients with resectable PDAC.

**Methods:**

We analyzed public datasets to compare GFPT1 expression in tumor tissues and normal/adjacent pancreatic tissues. We measured the relative GFPT1 expression of 134 resected PDAC specimens in our institution, using real-time polymerase chain reaction (PCR). Survival was compared between high and low GFPT1 expression groups using Kaplan-Meier curves and log-rank tests. Multivariate analyses were estimated using Cox regression and logistic regression models.

**Results:**

GFPT1 is generally upregulated in PDAC tissues, according to the analysis of public datasets. The data from our institution shows that high GFPT1 expression was correlated with a high rate of lymph node (LN) metastasis (*p* = 0.038) and was an independent risk factor for LN metastasis (odds ratio (OR) = 3.14, 95% confidence interval (CI) = 1.42 to 6.90, *P* = 0.005). High GFPT1 expression was significantly associated with poor overall survival (OS; *P* = 0.019) in patients with resected PDAC. The multivariable-adjusted hazard ratio (HR) for mortality when comparing patients with high and low GFPT1 expression was 2.54 (95% CI = 1.35 to 4.79, *P* = 0.004).

**Conclusions:**

GFPT1 is generally upregulated in PDAC tissue and is associated with a high risk of LN metastasis and an unfavorable outcome in patients with resectable PDAC, suggesting its crucial role in PDAC progression.

## Introduction

Pancreatic ductal adenocarcinoma (PDAC) is one of the most aggressive malignancies with a dismal survival rate and is estimated to become the second leading cause of cancer-related death in the USA by 2030 [1]. Despite the relentless effort during the past decades, the survival rate of PDAC has only marginally improved, and the 5-year survival rate remains 9%, the lowest among cancers [2]. Surgery remains the only potential way to cure PDAC. However, since early-stage PDAC generally lacks clinical symptoms, less than 20% of patients are eligible for initial surgical resection, as they are diagnosed at a late stage [3, 4]. Nevertheless, nearly 80% of PDAC patients experience local recurrence or distant metastases within 2 years of a potential curative resection, mostly because of the presence of micrometastases before the initial resection [5]. Hence, apart from surgical resection, comprehensive treatment following surgery is also required. In addition to well-established therapies, including chemotherapy and radiotherapy, targeted therapy has emerged as a prospective anticancer regimen [6–8]. Disappointingly, most clinical trials on targeted therapy exhibit unsatisfactory outcomes [9]. Therefore, exploration of new targets in PDAC treatment is urgently needed.

Reprogramming energy metabolism is considered one of the core hallmarks of cancer [10]. The aberrant metabolism of glucose is one of the most prominent characteristics. As a branch of the glucose metabolic pathway, the hexosamine biosynthesis pathway (HBP), though merely constituting 2–5% of the total glucose consumption, plays a pivotal role in driving tumorigenesis and promoting cancer proliferation. Moreover, the end product of HBP, uridine diphosphate N-acetylglucosamine (UDP-GlcNAc), an essential substrate for N-linked or O-linked protein glycosylation, is an important regulator of cell signaling and may affect various functional targets that ultimately influence cancer phenotypes [11]. Furthermore, apart from glucose, the HBP also utilizes glutamine, another fundamental nutrient essential in cell growth. Glutamine is catalyzed by the first rate-limiting enzyme in HBP, which is called glutamine-fructose-6-phosphate transaminase 1 (GFPT1) and is eventually transformed into UDP-GlcNAc. Both glutamine and glucose anabolism are vital to pancreatic cancer survival and growth [12]. Therefore, we assume that GFPT1, an enzyme that not only integrates glucose and glutamine metabolism but also influences protein glycosylation in key signaling pathways, may be a critical regulator in PDAC progression. Previous studies reported that high expression of GFPT1 correlated with unfavorable prognosis in hepatocellular carcinoma [13]. A recent study suggested that inhibiting GFPT1 expression via pharmaceutical inhibitors or genetic silencing may result in reduced cell proliferation and cancer invasiveness [14]. However, the correlation between GFPT1 gene expression and clinical outcomes of resectable PDAC patients remains to be elucidated.

In this study, we used quantitative polymerase chain reaction (qPCR) to assess the expression of GFPT1 in PDAC tissues in patients who underwent surgical resection and its correlation with clinicopathologic characteristics and postoperative survival of patients. We also analyzed The Cancer Genome Atlas (TCGA) pancreatic adenocarcinoma (PAAD) database, different Gene Expression Omnibus (GEO) datasets, and the Genotype-Tissue Expression (GTEx) project (pancreas dataset) to validate our findings.

## Methods

### Patients and specimens

Tumor specimens consisted of 134 pancreatic cancer tissues obtained from patients who underwent radical surgical resection and were pathologically diagnosed with pancreatic ductal adenocarcinoma from 2012 to 2016 in the Department of Pancreatic Surgery Shanghai Cancer Center, Fudan University, China. The clinicopathological and baseline characteristics of the patients, including age, sex, tumor size, tumor site, tumor differentiation, tumor stage, status of perineural infiltration, vessel invasion, nodal metastasis, and pre-operative carbohydrate antigen 199 (CA199) level, were collected from the patients’ medical history from Shanghai Cancer Center. Tumor stage was histologically determined based on the tumor-node-metastasis (TNM) staging for pancreatic cancer from the American Joint Committee on Cancer (AJCC), 8th edition [15]. Tumor size was based on the histological pathology report of resectable specimens. Overall survival (OS) was defined as the length of time (days) from the beginning of treatment to death from any cause (or the last follow-up). Follow-up ended in November 2019. This study was approved by the Ethics Board of Shanghai Cancer Center, Fudan University, and all patients involved in this study provided informed consent for the use of their personal data for research purposes.

### TCGA, GEO, and GTEx datasets

The data are publicly available from TCGA (PAAD dataset) and the GEO database (accession number: GSE28735, GSE62542, GSE16515, GSE15471) and the GTEx project (pancreas dataset). Level 3 gene expression data of PAAD patients (fragments per kilobase of transcript per million mapped reads [FPKM] normalized) and the corresponding clinical parameters in the TCGA were downloaded from the UCSC Xena browser (https://xenabrowser.net). The mRNA expression in the TCGA dataset was assayed by RNA sequencing V2. For the relative GFPT1 mRNA expression comparison between PDAC and normal pancreas, the figure was generated from the Gene Expression Profiling Interactive Analysis 2 (GEPIA2) database (http://gepia2.cancer-pku.cn), whose RNA-Seq datasets are based on the UCSC Xena project (http://xena.ucsc.edu) [16]. For the GEO dataset, the relative mRNA expression of GFPT1 was acquired and analyzed through GEO2R and R (version 4.0.2) with the “GEOquery” package.

### Real-time PCR

Total RNA was extracted from PDAC tissues using the Tissue RNA Purification Kit PLUS (Cat# EZB-RN001-plus, EZBioscience) following the manufacturer’s instructions. RNA reverse transcription to cDNA was conducted using 4× EZscript Reverse Transcription Mix II (Cat# EZB-RT2GQ, EZBioscience) according to the manufacturer’s protocols. Real-time PCR was performed using a QuantStudio™ 7 Flex Real-Time PCR system (Cat# 4485701, Applied Biosystems). ACTB (β-actin) was used as an internal control. The relative mRNA expression of GFPT1 was normalized to ACTB expression. All reactions were run in triplicate. The primer sequences were as follows: GFPT1 forward, 5-TTGCCTGTGATGGTGGAACT-3; GFPT1 reverse, 5-GTGATATGGAACTGCCAACTGT-3; ACTB forward, 5-CGTGCGTGACATTAAGGAAGAGT-3; and ACTB reverse, 5-GGAAGGAAGGCTGGAAGAGT-3.

### Statistical analysis

Receiver operating characteristic (ROC) curve analysis was performed to determine the optimal cutoff value of GFPT1 mRNA expression to stratify the patients into low and high GFPT1 expression groups. Pearson’s *χ*^2^ tests and Fisher’s exact tests were used to analyze the correlation between GFPT1 expression and major baseline characteristics. Survival curves of different groups were compared using log-rank tests. The results of the multivariate analyses were estimated with Cox regression models and logistic regression models. Student’s two-tailed *t* test was used to compare differences between two groups; the Mann-Whitney test was performed when the normality test (*α* = 0.05) was not passed. Differences with *p* values less than 0.05 were considered statistically significant, and all *p* values are two-sided. All statistical analyses were performed using the SPSS 26.0 software and R software 4.0.2.

## Results

### GFPT1 expression is upregulated in PDAC

**Fig. 1:**
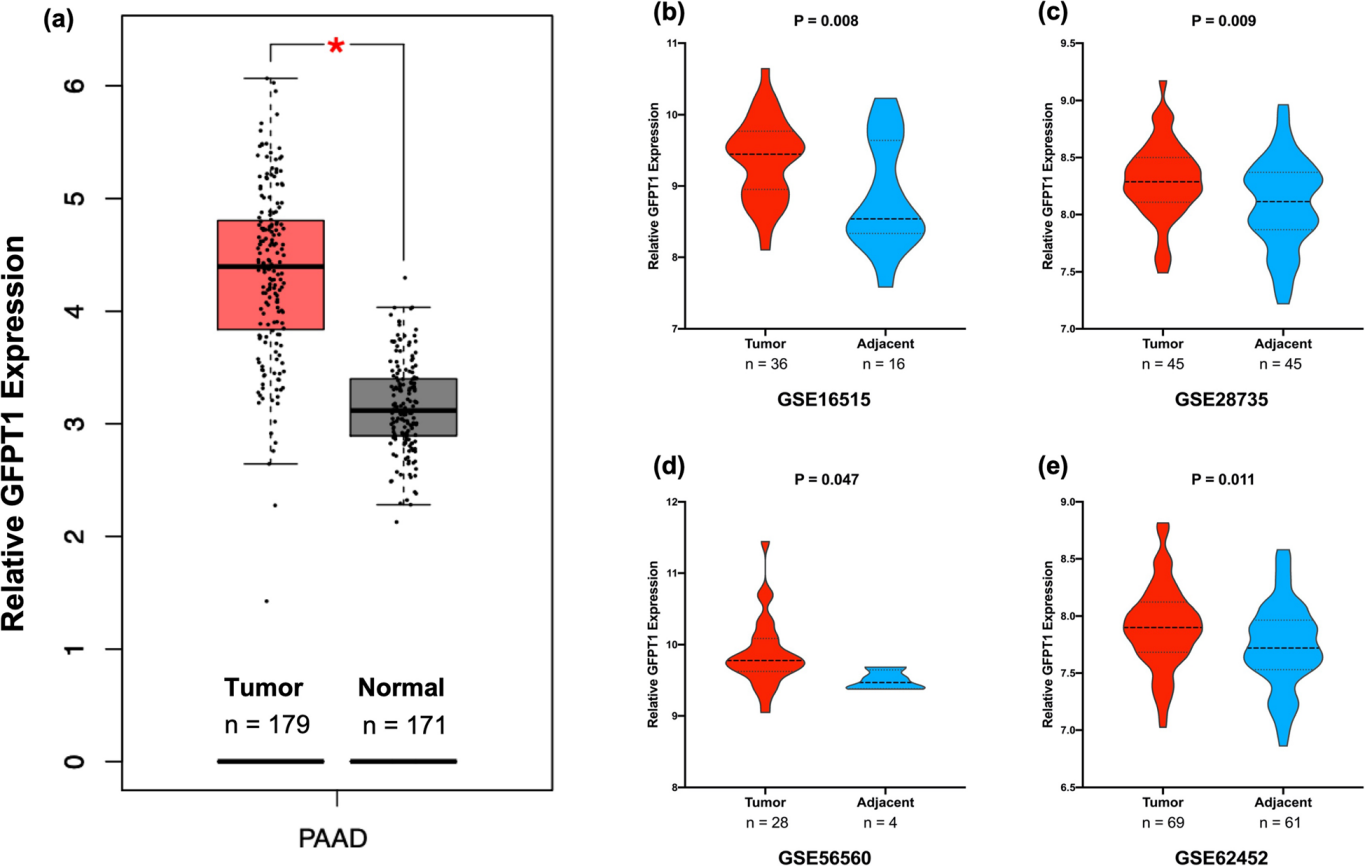
GFPT1 expression pattern in public datasets (**a**). Comparisons of relative GFPT1 mRNA expression between pancreatic ductal adenocarcinoma tissue (from TCGA PAAD datasets) and normal pancreas tissue (from GTEx datasets) were generated at http://gepia2.cancer-pku.cn [16] (**b**–**e**). Comparisons of relative GFPT1 mRNA expression between pancreatic ductal adenocarcinoma tissue and adjacent nontumor tissue from GSE16515 (**b**), GSE28735 (**c**), GSE56560 (**d**), and GSE62452 (**e**). Comparisons (**b**, **c**, and **e**) were conducted by performing Student’s two-tailed *t* test. Comparison (**d**) was conducted by performing the Mann-Whitney test because the normality test was not passed. GFPT1, glutamine-fructose-6-phosphate transaminase 1

To investigate the role of GFPT1 in pancreatic tumorigenesis, we examined the mRNA expression levels of GFPT1 in pancreatic tumor and normal tissues by analyzing GEO, TCGA, and GTEx dataset analyses. As is shown in Fig. 1, we found that GFPT1 expression at the mRNA level was increased in tumor tissues compared to adjacent nontumor tissues in GSE16515 (*P* = 0.008), GSE28735 (*P* = 0.009), GSE56560 (*P* = 0.047), and GSE62452 (*P* = 0.011), and GFPT1 mRNA expression in pancreatic cancer tissues was also higher than that in normal pancreatic tissues according to analysis integrating TCGA PAAD and GTEx datasets (*P* < 0.001).

### Association between GFPT1 levels and clinicopathological characteristics in resectable PDAC patients

To investigate the clinicopathological significance of GFPT1 mRNA expression in PDAC tissues, we assayed the relative GFPT1 expression using quantitative PCR and further assessed the relationship between GFPT1 expression and clinicopathological characteristics in 134 resectable PDAC samples who all underwent radical surgical resection. The cutoff value of GFPT1 expression defined by ROC curve analysis was 0.02145, which stratified patients into those with high GFPT1 expression (*n* = 67) and those with low GFPT1 expression (*n* = 67) using. The correlation between GFPT1 expression and baseline characteristics in PDAC patients was analyzed with Pearson’s *χ*^2^ tests or Fisher’s exact tests, and the results are shown in Table 1. Patients with higher GFPT1 expression were more likely to have nodal metastasis at the initial resection (*P* = 0.038). The distributions of age, sex, tumor location, tumor grade, tumor stage, perineural infiltration, vessel invasion, and pre-operative CA199 level were not significantly different between the two groups.

**Table 1:** Correlation between GFPT1 expression and clinicopathological variables

Variables	GFPT1 expression		P value
	Low (***n*** = 67)	High (***n*** = 67)	
Age, *n* (%)			
< 60 years	29 (43.3%)	35 (52.2%)	0.299
≥ 60 years	38 (56.7%)	32 (47.8%)	
Sex, *n* (%)			
Male	32 (47.8%)	40 (59.7%)	0.166
Female	35 (52.2%)	27 (40.3%)	
Tumor location, *n* (%)			
Head and neck	39 (58.2%)	32 (47.8%)	0.229
Body and tail	28 (41.8%)	35 (52.2%)	
Tumor grade, *n* (%)			
High and intermediate	40 (59.7%)	42 (62.7%)	0.723
Low	27 (40.3%)	25 (37.3%)	
Tumor size, *n* (%)			
≤ 4.0 cm	53 (79.1%)	49 (73.1%)	0.418
> 4.0 cm	14 (20.9%)	18 (26.9%)	
Nodal metastasis, *n* (%)			
Absence	40 (59.7%)	28 (41.8%)	0.038*
Presence	27 (40.3%)	39 (58.2%)	
Perineural infiltration, *n* (%)			
Absence	10 (89.1%)	13 (77.6%)	0.492
Presence	57 (10.9%)	54 (22.4%)	
Vessel invasion, *n* (%)			
Absence	51 (76.1%)	57 (85.1%)	0.190
Presence	16 (23.9%)	10 (14.9%)	
CA199 level, *n* (%)			
≤ 37 U/ml	26 (38.8%)	19 (28.4%)	0.200
> 37 U/ml	41 (61.2%)	48 (71.6%)	

*GFPT1* glutamine-fructose-6-phosphate transaminase 1, *CA199* carbohydrate antigen 199

*P value < 0.05

### High GFPT1 expression is an independent risk factor for nodal metastasis

**Fig. 2:**
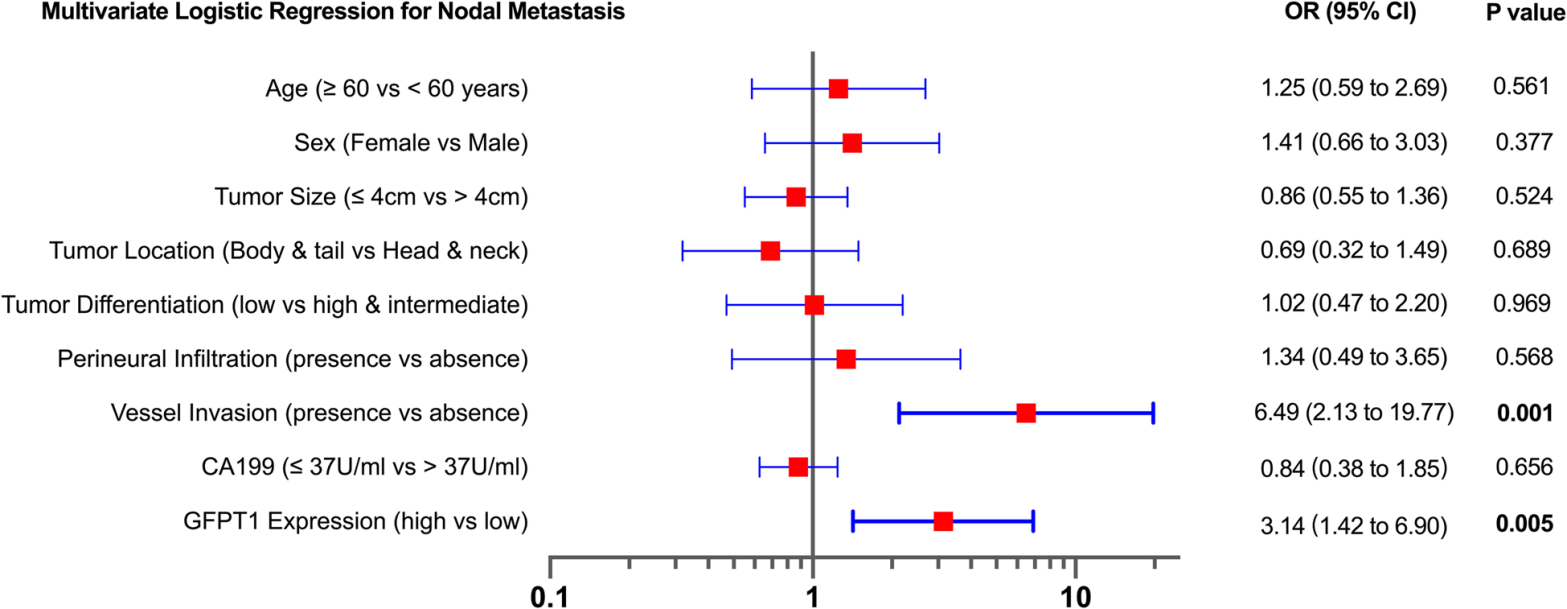
Logistic regression analysis for LN metastasis. Multivariate analysis for nodal metastasis was performed using a logistic regression model. The presence of vessel invasion and high GFPT1 expression were found to be independently associated with nodal metastasis. OR, odds ratio; GFPT1, glutamine-fructose-6-phosphate transaminase 1; CA199, carbohydrate antigen 199

To examine whether high GFPT1 expression was independently associated with nodal metastasis, multivariate analysis was conducted using logistic regression after adjusting for major pathological features, including tumor location, tumor grade, tumor size, perineural infiltration, vessel invasion, and pre-operative CA199 level (Fig. 2). The analysis showed that high GFPT1 expression was independently correlated with nodal metastasis (odds ratio (OR) 3.14, 95% CI 1.42 to 6.90, *p* = 0.005). Vessel invasion was also shown to be independently associated with nodal metastasis (OR = 6.49, 95%CI = 2.13 to 19.77, *p* = 0.001).

### Correlations between GFPT1 expression and overall survival (OS) in PDAC patients

**Fig. 3:**
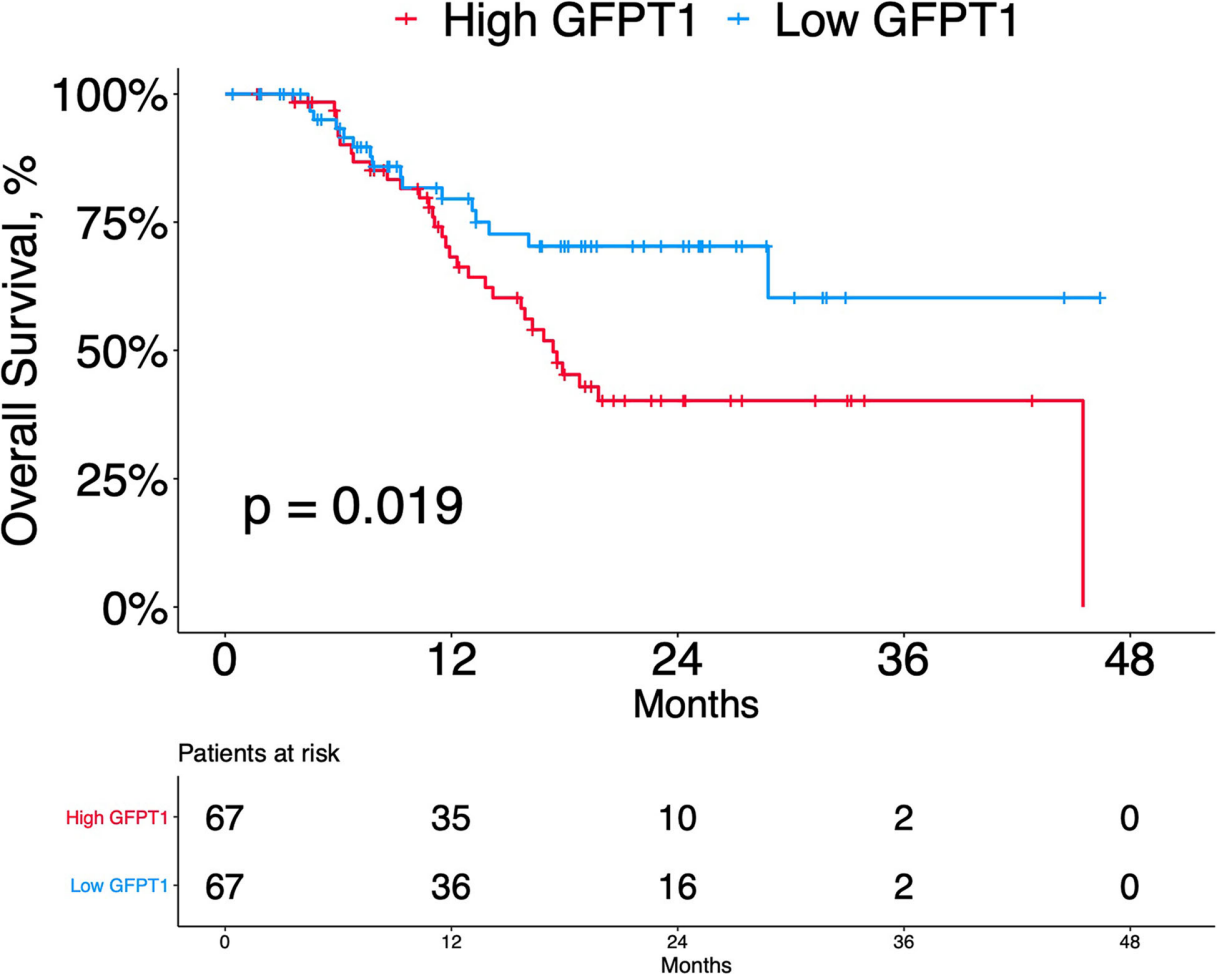
Kaplan-Meier curves for OS Kaplan-Meier curves for OS comparing PDAC patients with high (*n* = 67) and low (*n* = 67) GFPT1 expression. Cutoff values were determined by ROC curve analysis. *P* value was derived from log-rank test. GFPT1, glutamine-fructose-6-phosphate transaminase 1

We next evaluated the correlation between GFPT1 expression and survival in PDAC patients via Kaplan-Meier analyses (Fig. 3). The median follow-up (range) for the patients of the study was 19.4 (1.4 to 46.4) months. The results of the log-rank test suggested that high GFPT1 expression indicated worse OS of resectable PDAC patients (*P* = 0.019). The median survival time for those with high GFPT1 expression was 17.4 months (95% CI 14.4 to 20.4 months), and the median survival time for those with low GFPT1 expression was not reached. Mortality rates for patients with high and low GFPT1 expression were 47.8% and 23.9%, respectively.

### GFPT1 expression is indicated as an independent predictor in patients with PDAC

**Fig. 4:**
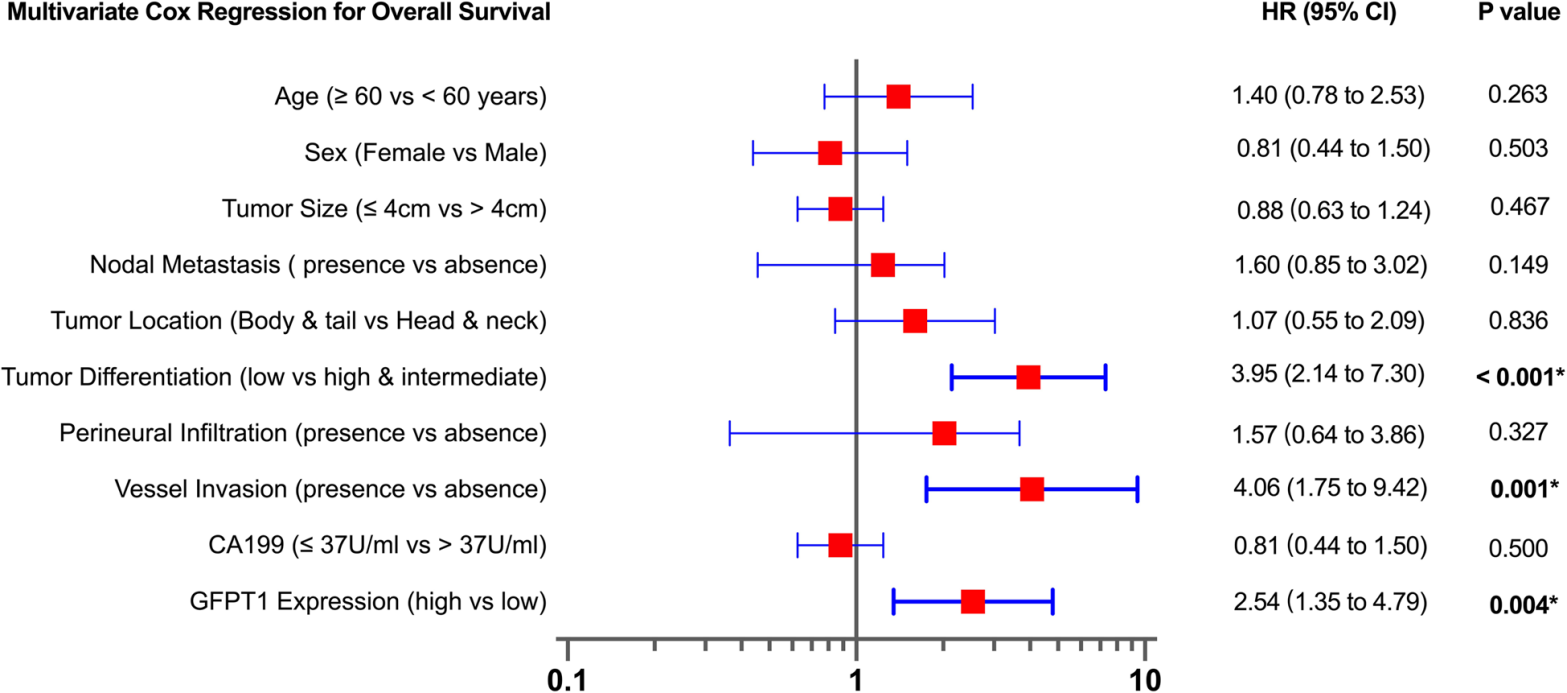
Cox regression analysis for mortality. Multivariate analysis of mortality was performed by using the Cox regression model. Low tumor differentiation, the presence of vessel invasion, and high GFPT1 expression were found to be independently associated with a high risk of mortality. HR, hazard ratio; GFPT1, glutamine-fructose-6-phosphate transaminase 1; CA199, carbohydrate antigen 199

In the multivariable-adjusted Cox regression model (Fig. 4) that included age, sex, tumor location, tumor size, tumor differentiation, vessel invasion, perineural infiltration, and pre-operative CA199 level, the HR for mortality comparing those with high and low expression of GFPT1 was 2.54 (95% CI = 1.35 to 4.79, *P* = 0.004), suggesting that high expression of GFPT1 is independently associated with OS following surgical resection in PDAC patients. Presence of vessel invasion and low tumor differentiation were also independently correlated with worse OS, and the adjusted HR for mortality was 4.06 (95% CI = 1.75 to 9.42, *P* = 0.001) and 3.95 (95% CI = 2.14 to 7.30, *P* < 0.001).

## Discussion

Pancreatic ductal adenocarcinoma is a representative aggressive malignancy that survives and thrives in a metabolic milieux characterized by poor nutrients and severe hypoxia. PDAC overcomes the harsh environment through rewiring metabolic pathways and employing unconventional approaches to acquire and utilize fuel sources [17], which also contributes to the malignant phenotype of PDAC. Glucose and glutamine serve as two predominant fuel sources for ATP generation and macromolecular biosynthesis, sustaining and facilitating the unlimited growth and proliferation of cancer cells [17]. The HBP pathway, though being a shunt pathway of glycolysis, also integrates glutamine metabolism, which is catalyzed by the first rate-limiting enzyme, GFPT1, and ultimately produces UDP-GlcNAc, a crucial donor for the O- and N-glycosylation of proteins, which plays critical roles in tumor progression. Therefore, we hypothesize that the expression level of GFPT1 may predict the prognosis of resectable PDAC patients. We analyzed public data from TCGA, GEO, and GTEx to assess the prognostic value of GFPT1 and further corroborate its significance by measuring the relative mRNA level of GFPT1 in resectable PDAC samples in our clinical center and investigated the correlation between GFPT1 expression and clinicopathological variables and the clinical outcome of patients.

In this study, we found that GFPT1 is generally elevated in PDAC, according to the data obtained from public datasets. After measuring the relative mRNA level of GFPT1 using a real-time PCR assay in 134 resectable PDAC samples and comparing the data with clinicopathological characteristics and postoperative outcomes, we found that a high GFPT1 level is associated with a high risk of LN metastasis and is an independent risk factor for nodal metastasis. A high GFPT1 level also indicates unfavorable postoperative OS and may independently predict clinical outcome. Demonstrating high GFPT1 expression as an independent prognostic factor, we believe, may provide new insights in the predicting of outcome of PDAC because conventional prognostic factors such as tumor differentiation, tumor size, and LN involvement fail to take account of the essential molecular and cellular mechanisms during the oncogenesis and progression of PDAC, while GFPT1 expression, to a certain extent, reflects the flux of HBP pathway, which integrates glutamine and glucose metabolism and plays crucial roles in tumor progression. Therefore, GFPT1 expression may serve as a complementary factor to better predict the outcome of PDAC on a molecular basis. Moreover, the association we have found between GFPT1 expression level and lymph node involvement does not only help the prediction of nodal metastasis, but more importantly, indicated via which mechanism GFPT1 upregulation may cause worse survival in PDAC. Since nodal involvement is a very significant process of tumor progression, it is helpful to know that GFPT1 may independently contribute to nodal metastasis. More importantly, the study may also provide novel understanding in the mechanism of PDAC progression. PDAC is a well-known glutamine-addicted cancer and is extremely sensitive to glutamine deprivation [18]. GFPT1/HBP functions as a coordinator of glucose and glutamine metabolism and is upregulated in various types of cancer [14]. A recent study indicated that oncogenic KRAS signaling, induced by a signature mutation in PDAC, markedly augmented the flux of HBP by inducing the expression of GFPT1 [19]. Moreover, GFPT1/HBP is also suggested to be enhanced in hypoxia [20], which is a quintessential characteristic of the PDAC microenvironment. These findings explain the upregulation of GFPT1 in PDAC tissues. It has also been suggested that knockdown of GFPT1 in PDAC cell lines inhibits carcinogenic activity both in vitro and in vivo, demonstrating the critical role of GFPT1 in the maintenance and progression of PDAC [19]. GFPT1 was also found to induce the aggressive biology of PDAC by regulating self-renewal genes and by facilitating cancer cell invasion and migration [21]. GFPT1 also plays an instrumental role in shaping the tumor immune microenvironment in PDAC, contributing to an immunosuppressive immune milieu, probably through modulating the extracellular matrix of cancer cells [21]. It was also reported that cells may be sensitized to anti-PD1 immunotherapy when GFPT1 is inhibited [21]. These results consistently suggest that high GFPT1 expression is associated with a poor prognosis in PDAC and furthermore indicate GFPT1 as a promising target for antitumor therapy in PDAC.

The major strength of our study is that it focused mainly on resectable PDAC patients who underwent radical surgery, which improved the homogeneity of the cohort we studied. Moreover, we integrated public datasets and data from our institution, which increases the validity. Further, we measured the expression of GFPT1 at the mRNA level by real-time qPCR, which is more precise than immunohistochemical (IHC) staining, because IHC staining results are usually presented as a spectrum of categories which describe different intensities of IHC expression as “negative,” “weak,” “moderate,” “strong,” or other variations, instead of specific value as quantitative PCR, and the interpretation of the results are relatively subjective due to the lack of scoring standard for IHC staining of GFPT1 [22]. The major limitations of our study are its retrospective nature and the relatively small size of the PDAC patient cohort. Additionally, crucial information, such as nutritional status, postoperative complications, specific cause of death, and status of adjuvant chemo/radiotherapy, was not included in our data, which may compromise the validity of our results. In addition, well-established prognostic variables, including nodal metastasis and perineural infiltration, were found to be statistically nonsignificant in the multivariate analysis of OS, which also presents questions about the validity. Moreover, the study mainly focused on the mRNA level of GFPT1 expression and failed to investigate the significance of post-transcriptional level of GFPT1. However, we believe this has provided another perspective in understanding the prognostic role of GFPT1. Prospective studies in larger cohorts with more detailed clinical characteristics should be performed to corroborate our findings. Further studies on the specific mechanisms by which GFPT1 upregulation may promote the progression and invasiveness of PDAC are also warranted.

In conclusion, GFPT1, as a rate-limiting enzyme in the HBP, plays a pivotal role in PDAC progression. We found that GFPT1 is generally upregulated in PDAC tissues compared to normal tissues and that higher GFPT1 expression is associated with a higher risk of LN metastasis. A high GFPT1 level also indicates poor postoperative OS and may independently predict clinical outcome. Further study of the mechanism by which GFPT1 promotes PDAC progression is needed.

## Data Availability

Not applicable.

## Abbreviations

PDAC: Pancreatic ductal adenocarcinoma
HBP: Hexosamine biosynthesis pathway
UDP-GlcNAc: Uridine diphosphate N-acetylglucosamine
GFPT1: Glutamine-fructose-6-phosphate transaminase 1
qPCR: Quantitative polymerase chain reaction
TCGA: The Cancer Genome Atlas
PAAD: Pancreatic adenocarcinoma
GEO: Gene Expression Omnibus
GTEx: Genotype-Tissue Expression
TNM: Tumor-node-metastasis
AJCC: American Joint Committee on Cancer
FPKM: Fragments per kilobase of transcript per million mapped reads
ROC: Receiver operating characteristic
CA199: Carbohydrate antigen 199
OR: Odds ratio
HR: Hazard ratio
IHC: Immunohistochemistry
